# L-Quebrachitol Attenuates RANKL-Induced Osteoclastogenesis and Bone Resorption in Ovariectomized Rat Model

**DOI:** 10.3390/biom16010168

**Published:** 2026-01-20

**Authors:** Purithat Rattajak, Aratee Aroonkesorn, Thanintorn Yodthong, Acharaporn Issuriya, Siriluk Maskaew, Carl Smythe, Rapepun Wititsuwannakul, Thanawat Pitakpornpreecha

**Affiliations:** 1 Division of Health and Applied Science (Biochemistry), Faculty of Science, Prince of Songkla University, Hat-Yai 90110, Songkhla, Thailand; 2 Center of Excellence for Biochemistry, Faculty of Science, Prince of Songkla University, Hat-Yai 90110, Songkhla, Thailand; 3 Center for Natural Rubber Latex Biotechnology Research and Innovation Development, Prince of Songkla University, Hat-Yai 90110, Songkhla, Thailand; 4 Division of Health and Applied Science (Physiology), Faculty of Science, Prince of Songkla University, Hat-Yai 90110, Songkhla, Thailand; 5 Department of Biomedical Science, University of Sheffield, Sheffield S10 2TN, UK

**Keywords:** L-quebrachitol, osteoporosis, osteoclast, NF-κB, NFATc1, ovariectomized rat

## Abstract

Inositol is a natural carbocyclic sugar that plays an essential role in regulating the vital cellular functions of plants and animals. Existing research has explored methyl derivatives of inositol, reporting on their various biological activities, including antitumor, anti-inflammatory, and anti-osteoporosis activities. Our previous study demonstrated that L-quebrachitol, a methyl derivative of inositol, enhances osteoblastogenesis and bone formation; however, its effect on osteoclastogenesis remains unclear. Consequently, we aimed to investigate the effect of L-quebrachitol on receptor activator of nuclear factor-κB ligand-induced osteoclastogenesis in pre-osteoclastic RAW 264.7 cells, and bone resorption in an ovariectomized rat model. The results revealed that L-quebrachitol suppressed RANK-mediated signaling, including nuclear factor kappa-light-chain-enhancer of activated B cells (NF-κB) and Fos proto-oncogene (cFOS) pathways, at both the gene and protein levels. Moreover, the critical transcription factor for osteoclastogenesis, nuclear factor of activated T cells c1 (NFATc1), was downregulated. Inhibition of osteoclast-associated marker genes encoding proteolytic enzymes, such as tartrate-resistant acid phosphatase (TRAP), matrix metallopeptidase 9 (MMP-9), and cathepsin K, led to reduced formation of TRAP-positive multinucleated cells and resorption pits. In addition, proteasome subunit alpha type-5 (PSMA5), which is involved in the degradation of the NF-κB inhibitor, was also suppressed. In particular, the animal study clearly supported the bone homeostasis property of the agent by increasing the BV/TV (bone volume/total volume) and Tb.Th (trabecular thickness) in ovariectomized rats. These findings demonstrate the dose-dependent inhibitory effect of L-quebrachitol on osteoclastogenesis through the modulation of RANK-mediated signaling pathways and prevention of bone loss in an animal model. However, further exploration of the potential of L-quebrachitol as an effective approach for osteoporosis is required.

## 1. Introduction

Osteoporosis is a critical bone metabolic condition resulting from an imbalance between osteoblast-mediated bone formation and resorption, leading to bone loss [1,2,3]. It primarily affects elderly adults and is characterized by deteriorating bone architecture and increased fracture risk. In particular, excessive osteoclast-mediated bone resorption is considered a significant contributor to the onset and progression of osteoporosis. Treatment primarily involves the use of osteoclastogenesis inhibitors, such as bisphosphates, calcitonin, and estrogens [4,5]. Osteoclastogenesis is the development of osteoclasts from hemopoietic progenitor cells, regulated by cytokines and hormones [6]. Macrophage colony-stimulating factor (M-CSF) and receptor activator of nuclear factor-κB ligand (RANKL) play crucial roles in this process. M-CSF promotes the survival and proliferation of osteoclast precursors, while RANKL governs their differentiation and bone resorption [7,8]. Inhibition of RANKL prevents osteoporosis and bone loss [9]. During osteoclast development, RANKL binds to its receptor, RANK, and activates signaling pathways, including nuclear factor-kappa B (NF-κB), mitogen-activated protein kinase (MAPK), phosphoinositide 3-kinase (PI3K), and the calcium–calmodulin pathway, facilitated by the adaptor protein Tumor Necrosis Factor Receptor Associated Factor 6 (TRAF6) [6,10,11]. NF-κB is particularly recognized as a vital mechanism in osteoclastogenesis, and its deficiency reportedly leads to osteopetrosis and impaired osteoclast formation [12,13]. Activation of NF-κB and other transcription factors, such as Fos proto-oncogene (c-Fos) and nuclear factor of activated T cells cytoplasmic 1 (NFATc1), promotes the expression of essential enzymes involved in bone resorption by osteoclasts, including tartrate-resistant acid phosphatase (TRAP), cathepsin K, and matrix metallopeptidase 9 (MMP-9) [6].

L-Quebrachitol (2-O-methyl-L-inositol) is a methoxy analog of inositol. Inositol is a naturally occurring cyclitol with a six-hydroxylated carbon ring structure. It can be found in both free forms and as derivatives in plants, in phosphorylated forms (phytic acid) [14,15,16], and as a membrane phospholipid (phosphatidyl inositol) of the skeletal, heart, and reproductive tissues in mammals, including humans [17,18]. Myo-inositol and D-chiro-inositol are the most widely distributed forms in prokaryote and eukaryote cells. The methyl derivative has been extensively studied and reported to possess diverse biological activities, including protection from cardiovascular disease, in addition to anti-inflammatory, anticancer [19,20,21], and anti-osteoporosis activities. D-pinitol (3-O-methyl-D-chiro-inositol) is a methyl derivative of D-chiro-inositol. It has been reported to suppress osteoclast differentiation and alleviate bone loss in ovariectomized mice by inhibiting the NF-κB and MAPK pathways [22]. L-Quebrachitol is the isomer of D-pinitol found in several plants, including sea buckthorn (*Hippophae rhamnoides*) [23]. It is particularly abundant in *Hevea* latex serum, produced as a by-product from the rubber industry. Interestingly, it has been used as an optical starting material for the synthesis of various biologically active compounds such as antibiotics and enzyme inhibitors [23,24]. Moreover, L-Quebrachitol exhibits various pharmacological activities, including anti-platelet aggregation, platelet-activating factor (PAF) inhibition, gastroprotective effects, and antidiabetic properties [25,26,27]. Despite being an isomer of D-pinitol, L-Quebrachitol, like most isomers, displays significant differences in biological activities, such as toxicology, pharmacology, and metabolism [28]. A previous study clearly indicated that D-pinitol possesses inhibitory activity against osteoclastogenesis but has no effect on osteoblastogenesis [22]. Conversely, our study clearly demonstrated that L-quebrachitol enhances osteoblastogenesis through involvement in the BMP-2/Runx2/MAPK/Wnt/β-Catenin signaling pathway [29]. Furthermore, a recent study revealed that L-quebrachitol significantly augments bone mineral density and bone calcium content in ovariectomized rats [30]. Nevertheless, its effect on RANKL-induced osteoclastogenesis remains unclear.

Therefore, this study examined the impact and mechanism of L-quebrachitol on RANKL-induced osteoclastogenesis in the RAW 264.7 pre-osteoclastic cell line. Our findings demonstrate that L-quebrachitol effectively inhibits osteoclast proliferation, differentiation, and bone resorptive function. Moreover, bone resorption in the ovariectomized rat model was attenuated following oral administration of a low dose of quebrachitol. These results suggest that L-quebrachitol has potential as a natural compound for addressing bone metabolic issues.

Furthermore, these novel discoveries have the potential to broaden the utilization scope of L-quebrachitol and contribute to enhancing the value of natural latex rubber while mitigating environmental pollution. This aligns with Thailand’s national strategy of employing the integrated Bio-Circular-Green (BCG) Economic Model to propel the country’s economic and social advancement.

## 2. Materials and Methods

### 2.1. Materials

3-(4,5-dimethylthiazol-2-yl)-2,5-diphenyltetrazolium bromide (MTT) and D-Pinitol were purchased from Sigma Aldrich (St. Louis, MO, USA). M-CSF and RANKL were purchased from R&D Systems (Minneapolis, MN, USA). Antibodies against anti-β-actin (No. 4970S), anti-c-Fos (No. 2250S), anti-NF-κB-P65 (No. 8242S), anti-NFATc1 (No. 8032S), and anti-phospho-NF-κB-P65 (No. 3033S) were purchased from cell signaling technology (Danvers, MA, USA). L-Quebrachitol was obtained from the Center for Natural Rubber Latex Biotechnology Research and Innovation Development. A reference standard of L-quebrachitol (TRC-Q990090, purity > 95%) was obtained from Toronto Research Chemicals Inc. (Toronto, ON, Canada). Extraction and purification of L-quebrachitol were performed based on previously reported methods [29]. All other chemicals of analytical grade were purchased locally.

### 2.2. Extraction and Purification of L-Quebrachitol

L-quebrachitol was extracted and purified according to the method reported by Yodthong et al. (2018) [29], with minor modifications. Briefly, fresh latex (50 L) obtained from *Hevea brasiliensis* (RRIM 600 clone) was coagulated using 0.2% (*v*/*v*) formic acid, followed by separation of the serum fraction. The collected serum was sequentially concentrated via ultrafiltration using membranes with molecular weight cut-offs of 10 and 1 kDa. The resulting 1 kDa permeate was spray-dried and subsequently extracted with methanol at a ratio of 1:5 (*w*/*v*). After filtration and solvent evaporation, the crude crystalline material was recrystallized three times using 75% (*v*/*v*) aqueous ethanol. The recrystallized product was further purified through cation-exchange chromatography (Dowex 50W-X8 resin), followed by ethanol precipitation and freeze-drying to obtain purified L-quebrachitol. The purified compound was obtained as a freeze-dried powder and stored at −20 °C in airtight containers, protecting it from moisture until use. All subsequent biological experiments were performed using a single purification batch (Lot No. Qb231215) to minimize batch-to-batch variability.

### 2.3. Compound Identification and Purity Analysis

The identity of the purified compound was verified through a comparison with a commercial L-quebrachitol reference standard using high-performance size exclusion chromatography (HPSEC) and nuclear magnetic resonance (NMR) spectroscopy.

HPSEC analysis was performed on an Agilent 1260 Infinity II system (Agilent, Santa Clara, CA, USA) equipped with a quaternary pump, autosampler, column oven, and refractive index (RI) detector. Chromatographic separation was achieved using a PolySepTM P-2000 column (300 × 7.8 mm) coupled with a PolySep guard column (35 × 7.8 mm; Phenomenex, Torrance, CA, USA). Ultrapure water, filtered through a 0.45 μm membrane and degassed prior to use, served as the mobile phase. Isocratic elution was conducted at room temperature with a flow rate of 0.8 mL/min. All samples were prepared at a concentration of 10 mg/mL, filtered through a 0.45 μm membrane, and injected at a volume of 1 μL. The total run time for each analysis was less than 20 min.

Quantitative purity was determined by calculating the area under the curve (AUC) from the HPSEC chromatograms using an external calibration curve generated from the reference standard. Structural confirmation was further obtained via 1H and 13C NMR spectroscopy, performed using a Bruker AVANCE NEO 500 MHz NMR spectrometer at the Office of Scientific Instrument and Testing (OSIT), Prince of Songkla University, Songkhla, Thailand.

### 2.4. Cell Culture and Differentiation

The RAW 264.7 cell line was obtained from ATCC (Manassas, VA, USA). Cells were cultured in Dulbecco’s modified eagle’s medium (DMEM) containing 10% fetal bovine serum (FBS) and 1% penicillin/streptomycin. To induce osteoclast differentiation, cells were cultured in DMEM in the presence of 20 ng/mL of M-CSF and 20 ng/mL RANKL for 5 days.

### 2.5. Cell Viability Assay

The viability of RAW 264.7 cells was investigated using the MTT assay. We seeded RAW 264.7 cells in 96-well plates at a density of 5 × 10^3^ cells/well and incubated them with L-quebrachitol at various concentrations. After culturing for 24, 48, and 72 h, MTT (0.25 mg/mL) was added and then the samples were incubated at 37 °C for 3 h. To dissolve formazan products, DMSO was added and measured in a microplate reader (BioTek Instruments, Winooski, VT, USA) at 570 nm.

### 2.6. Osteoclast Differentiation Assay

To evaluate osteoclast formation, TRAP staining was performed. First, the cells were seeded into 96-well plates at a density of 1.4 × 10^3^ cells/well and incubated for 24 h. The cells were then incubated with induction medium for 5 days, prepared via the addition of 20 ng/mL of M-CSF and RANKL in the presence of L-quebrachitol at various concentrations. D-Pinitol (30 µM) was used as a positive control, and was previously shown to inhibit osteoclastogenesis through RANKL-induced p38, JNK, and NF-κB pathways [22]. Then, the cells were fixed with 4% paraformaldehyde for 10 min and TRAP staining was performed using TRAP staining kits (Sigma Aldrich). TRAP-positive multinucleated cells with at least three nuclei were observed via light microscopy (Olympus, Tokyo, Japan); captured areas were selected from the middle of the well in all experimental groups.

### 2.7. Pit Formation Assay

Bone resorption was determined using the pit formation assay. Briefly, cells were plated into osteoassay surface 96-well plates (corning osteoassay, Corning, NY, USA ) at a density of 1.4 × 10^3^ cells/well and treated with L-quebrachitol at various doses. After 7 days, the medium was aspirated and incubated with bleach solution (100 µL/well) for 5 min at 25 °C and then the plate was washed with distilled water and air-dried for 3–5 h. Three resorption pit areas/sample were captured using a light microscope and measured using Image J software (version 1.53).

### 2.8. Real-Time Quantitative Polymerase Chain Reaction

RAW 264.7 cells were seeded in inducing medium in a 6-well plate (at a density of 1.4 × 10^3^ cells/well) and treated with L-quebrachitol at various concentrations for 3 days. In this study, 2.5 µM of cells treated with BAY11-7082, an NF-κB inhibitor, was added to the experiments to compare the suppressive ability of L-quebrachitol on NF-κB-P65. Total RNA was extracted using TRIzol reagent (Thermo Fisher Scientific Inc., Waltham, UT, USA) according to the manufacturer’s protocol. Then, an oligo-dT primer and reverse transcriptase (Thermo Scientific Inc., Waltham, UT, USA) were used for the production of cDNA. Real-time PCR was performed using 5× HOT FIREPol^®^Blend Master Mix (Solis Biodyne, Tartu, Estonia). Thermal cycling was set at 95 °C for 15 min for initial denaturation, 40 cycles of denaturation at 94 °C for 15 s, annealing at 57 °C for 30 s, and final elongation at 72 °C for 30 s. The specific primers are shown in Table 1. The relative mRNA levels of target genes were analyzed using the 2^−ΔΔCT^ method with *GAPDH* as an internal control.

### 2.9. Western Blot Analysis

RAW 264.7 cells were seed into 6-well plates at a density of 1 × 10^5^ cells/well overnight and treated with various doses (0.00005–60 µM) of L-quebrachitol. In addition, the suppressive activity of L-quebrachitol on NF-κB-P65 was determined by culturing cells with 2.5 µM BAY11-7082. After 3 days, the cells were lysed in RIPA lysis buffer containing a proteinase and phosphatase inhibitor cocktail (Thermo Fisher Scientific Inc., Waltham, UT, USA). Then, 15 µg of protein/lane was separated on 12% SDS-PAGE and transferred onto PVDF membranes (Millipore, Jaffrey, NH, USA). Nonspecific binding was blocked with 5% non-fat dried milk for 1 h. Then, the membranes were incubated with a primary antibody (1:1000) at 4 °C. After washing with TBS-Tween 20, the membrane was incubated with horseradish peroxidase (HRP)-conjugated secondary antibodies (1:5000) (N0. 7074S, Cell Signaling Technology). The signal was detected using CL Substrate Kit (Thermo Fisher Scientific Inc., Waltham, UT, USA) with X-ray film exposure (Thermo Fisher Scientific Inc., Waltham, UT, USA) inside a dark room. The films were scanned using scanners and analyzed using Image J software to determine protein intensity. Western blot original images can be found in Supplementary Materials.

### 2.10. Immunofluorescence

An immunofluorescence assay was performed to investigate the expression of RANK. Cells were plated into 18-well chambered cover glass (2 × 10^4^ cells/well) and incubated with L-quebrachitol at various concentrations. To assess immunofluorescence, cells were washed with PBS, fixed with 4% paraformaldehyde, permeabilized in 0.1% Triton X-100, and then blocked with 5% goat serum. After washing with PBS, the cells were incubated with the anti-RANK antibody (1:500; No.sc-390655Santa Cruz Biotechnology), and then washed, incubated with the secondary antibody (1:200; No. sc-516141, Santa Cruz Biotechnology), and stained with DAPI (1 μg/mL) for 5 min. Finally, a drop of the mounting medium was carefully added, and then visualized using a fluorescence microscope (Olympus, Tokyo, Japan). Then, 3 images were captured and measured using Image J software. The percentage of fluorescence intensity was subsequently calculated with following equation: [Fluorescence intensity (Treated group)/ Fluorescence intensity (Control group)] × 100.

### 2.11. Proteomic Analysis

RAW264.7 cells were seeded onto a 6-well plate for 24 h. Then, the cells were treated with L-quebrachitol at 60 μM under the stimulation of 20 ng/mL of M-CSF and RANKL. After 72 h, cells were harvested for proteomics analysis.

To analyze the whole proteome, cell pellets were lysed twice with metal beads in 50 mM ABC buffer using Mixer mill 400 (Retsch, Haan, Germany) for 3 min. The concentration of protein was verified using Bradford Reagent. The proteins were normalized to 1.60 mg/mL and incubated with 10 mM DTT at 65 °C for 30 min. After that, 25 mM IAA was added and the samples were incubated for 20 min, followed by incubation with 0.5 µg of trypsin to digest the proteins. After incubation for 16 h, the reaction was stopped by adding 1% formic acid. Supernatant was collected via centrifugation and transferred to an LC-MS vial for LC-MS analysis (Agilent QTOF 6545XT).

### 2.12. Study of Osteoporosis in the Animal Model

The ovariectomized rat model was employed to investigate the effect of quebrachitol in preventing osteoporosis. Twenty mature female Wistar rats weighing between 350 and 480 g (35–40 weeks old) were obtained from Nomura Siam International Co., Ltd., Bangkok, Thailand. The experimental protocols employed in this study followed Good Laboratory Practice guidelines and were approved by the Animal Ethical Committee of Prince of Songkla University, Thailand (Ref. 87/2021). Rats were randomly separated into four groups (n = 5): a sham control group (bilateral laparotomy without removing the ovaries), an ovariectomized model group (OVX), and two OVX groups that were each given a different dosage of L-quebrachitol (25 and 50 mg/kg body weight/day). Twelve weeks following OVX surgery, the animals were euthanized to obtain the left femurs and the L5 vertebrae for determination of bone volume/total volume (BV/TV) and trabecular bone thickness (Tb.Th) using a Micro-CT machine (µ-CT 35, SCANCO Medical AG, Wangen-Brüttisellen, Switzerland). To evaluate serum estrogen levels, blood samples were harvested from the hearts of the rats through cardiac puncture. Then, serum estrogen levels were evaluated using the Chemiluminescent Microparticle Immunoassay (CMIA) methodology, following the Architect system by Abbott.

### 2.13. Statistical Analysis

The data were expressed as the mean ± standard error of the mean (SEM). Significance was determined via a one-way ANOVA using Duncan’s or Turkey’s multiple range test. The statistical significance of the data was determined as *p* < 0.05.

## 3. Results

### 3.1. Purification Yield, Identity, and Purity of L-Quebrachitol

**Figure 1:**
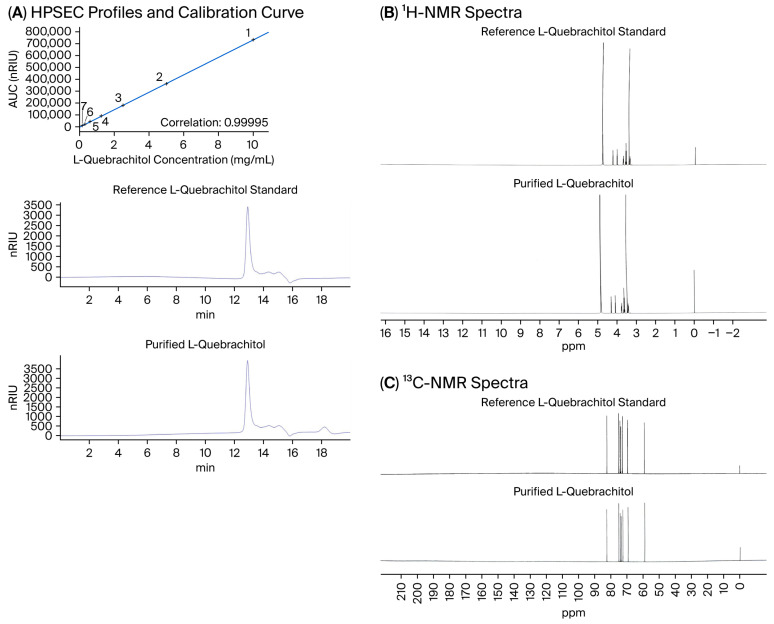
Structural characterization of purified L-quebrachitol isolated from fresh latex serum in comparison with a reference standard. (**A**) High-performance size-exclusion chromatography (HPSEC) elution profile of purified L-quebrachitol isolated from fresh latex serum compared with the calibration curve and a reference L-quebrachitol standard. (**B**) ^1^H NMR spectrum of purified L-quebrachitol isolated from fresh latex serum compared with the reference L-quebrachitol standard, recorded in D_2_O. (**C**) ^13^C NMR spectrum of purified L-quebrachitol isolated from fresh latex serum compared with the reference L-quebrachitol standard, recorded in D_2_O, confirming structural identity and purity.

The applied extraction and purification procedure yielded L-quebrachitol at 7.50 ± 2.11% (*w*/*w*) relative to the 1 kDa permeate powder. HPSEC analysis of the purified compound revealed a single peak at a retention time of 12.9 min, which was identical to that of the commercial L-quebrachitol standard (Figure 1A). Quantitative evaluation based on the AUC, determined using an external calibration curve exhibiting excellent linearity (R2 = 0.99995) (Figure 1A), indicated a high purity of the purified compound (98.12 ± 1.04%).

Structural identity was further confirmed via 1H and 13C NMR analyses, in which the purified L-quebrachitol displayed characteristic chemical shift patterns and signal distributions fully consistent with those of the reference standard (Figure 1B,C). Moreover, the purified L-quebrachitol was obtained as a stable, freeze-dried powder. No detectable degradation was observed during the experimental period, as verified by the consistent HPSEC chromatographic profiles obtained from the stored samples.

### 3.2. Toxicity Evaluation of L-Quebrachitol in RAW 264.7 Cells

**Figure 2:**
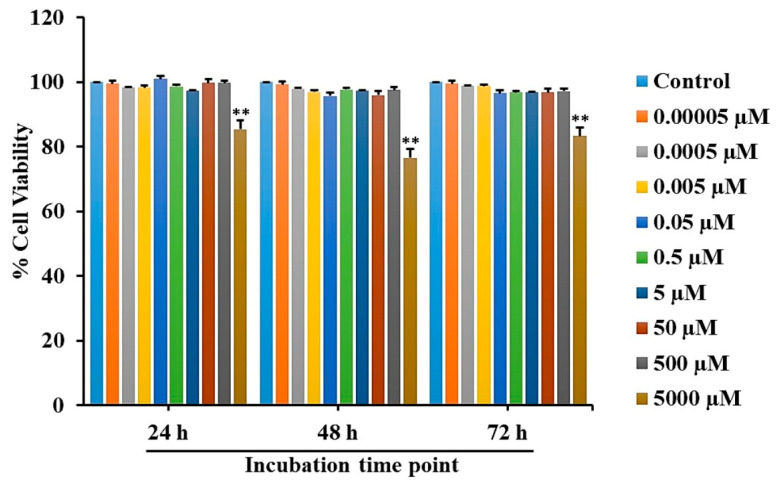
Cell viability evaluation of L-quebrachitol on RAW 264.7 cells. Cells were treated with varying concentrations of L-quebrachitol for 24, 48, and 72 h, and cell viability was detected with an MTT assay. Each data point represents the mean of 3 independent experiments on 4 identical samples ± SEM. ** *p* < 0.01 versus the control group.

To detect the cytotoxicity of L-quebrachitol, RAW 264.7 cells were treated with different concentrations of L-quebrachitol for 24, 48, and 72 h, followed by an MTT assay. The results revealed that when L-quebrachitol levels were lower than 500 µM, no significant change in cell viability was observed (Figure 2). Therefore, L-quebrachitol was used at 0.00005, 0.0005, 0.005, 0.05, 0.5, 5, 30, and 60 µM concentrations in further experiments.

### 3.3. Effect of L-Quebrachitol on Osteoclast Differentiation Induced by RANKL

**Figure 3:**
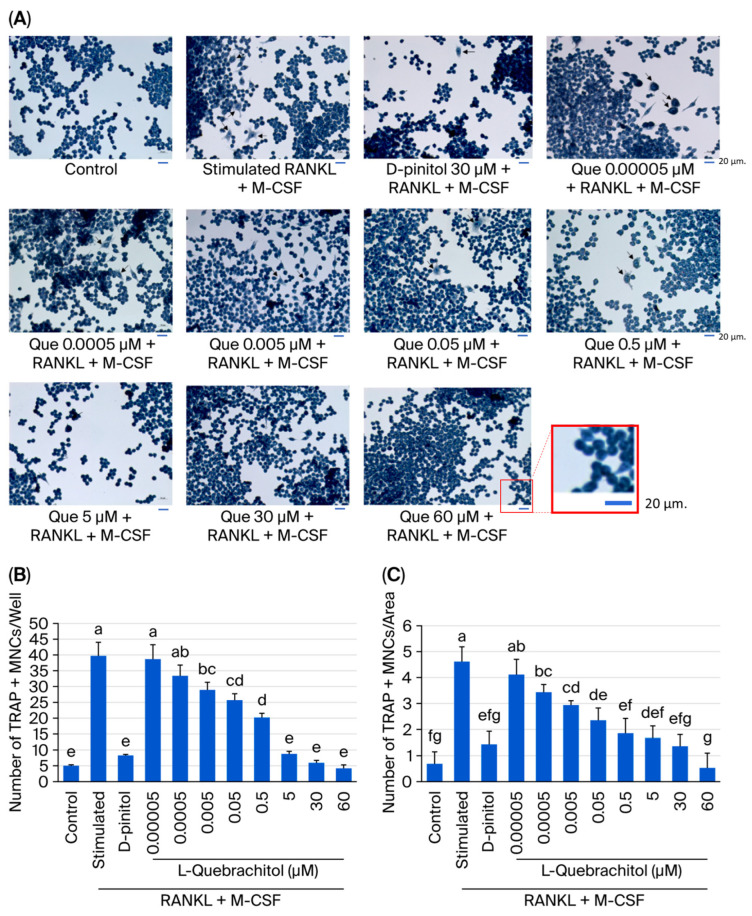
Effect of L-quebrachitol on multinucleated osteoclast formation, induced with RANKL. (**A**) RAW 264.7 cells were incubated with L-quebrachitol (0.05 nM–60 µM) under the stimulation of RANKL and M-CSF, and the number of differentiated cells, multinucleated osteoclasts, was observed using TRAP staining. Cells that consisted of at least three nuclei were specified as TRAP-positive and reported as (**B**) cells/well (96-well plate) and (**C**) cells/area. Each data point represents the mean from 3 independent experiments on 4 identical samples ± SEM. Letters on each column indicate statistically significant differences at *p* < 0.05.

We examined the inhibitory effect of L-quebrachitol on RANKL-induced osteoclast multinucleated cell formation. RAW 264.7 cells were treated with various dosages of L-quebrachitol (0.05 nM–60 µM) under stimulation with 20 ng/mL of RANKL and 20 ng/mL of M-CSF. The results of TRAP staining showed that L-quebrachitol inhibited the formation of mature osteoclasts from multinucleated cells into mature osteoclasts in a dose-dependent manner (Figure 3A–C). The TRAP-positive cell number in the RANKL and M-CSF stimulation group was 39 ± 2.6 cells/well or 3.9 cells/area, whereas that in the L-quebrachitol 60 µM-treated group decreased to 4.5 ± 0.6 cells/well or 0.5 cells/area, indicating the suppressive effect of L-quebrachitol on osteoclast formation (Figure 3B,C).

### 3.4. Effect of L-Quebrachitol on Osteoclast Bone Resorption Activity

**Figure 4:**
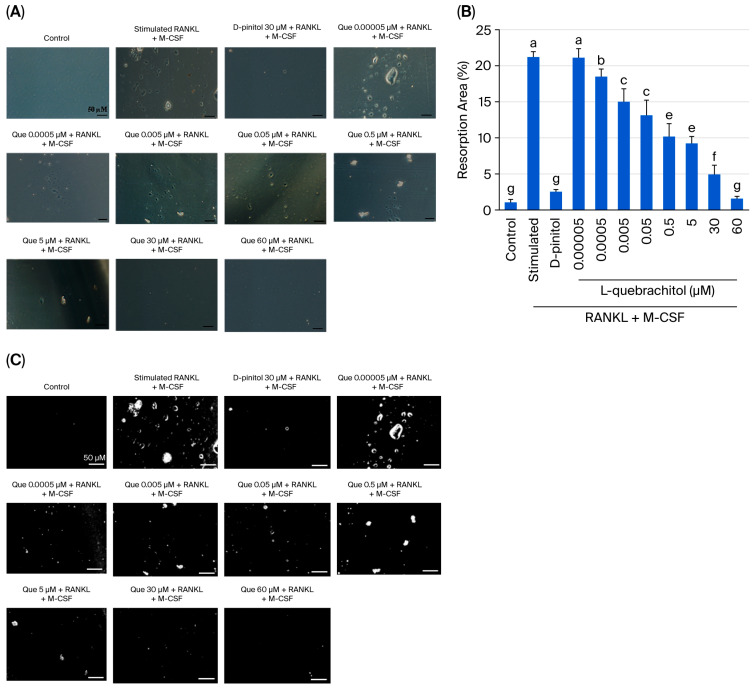
Effect of L-quebrachitol on the bone resorptive ability of osteoclasts. RAW 264.7 cells were incubated in an osteoassay 96-well plates and treated with RANKL (20 ng/mL), M-CSF (20 ng/mL) and different doses of L-quebrachitol for seven days. (**A**) Resorption pit areas observed under a microscope (scale bar = 50 μm) and (**B**) quantification and (**C**) analysis of bone resorption area with Image J. The data was obtained from 3 independent experiments on 4 replicates and presented as mean ± SEM. Letters on each column represent statistically significant differences at *p* < 0.05.

Because L-quebrachitol can suppress osteoclast differentiation in a dose-dependent manner, a pit formation assay was conducted in this experiment to detect osteoclastic resorptive ability. RAW 264.7 cells were cultured in a bone resorption assay plate, and after seven days, large areas of resorption were observed in the RANKL-stimulated group (Figure 4A–C). Conversely, the control group showed a smooth surface. Low concentrations of L-quebrachitol (0.005 µM) showed a significant decrease in pit formation area compared to that in the RANKL and M-CSF stimulation group (Figure 4A–C). Moreover, in the L-quebrachitol-treated group (60 µM), the bone resorption area was significantly reduced (Figure 4A–C). The results revealed that L-quebrachitol attenuated the bone resorption potential of osteoclasts.

### 3.5. L-Quebrachitol Downregulates mRNA Expression of NF-κB-P65, NFATc1, and C-Fos

**Figure 5:**
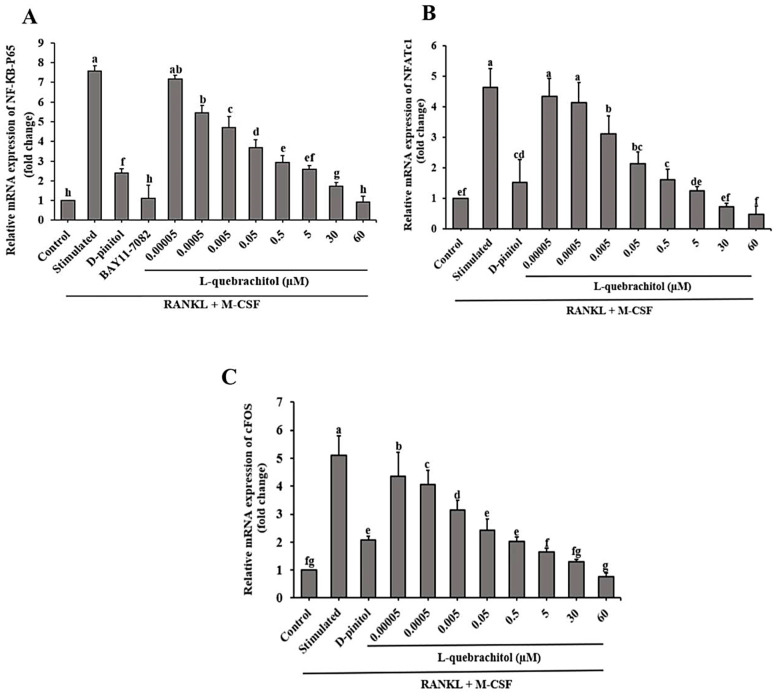
Effect of L-quebrachitol on the RANKL-induced NF-κB signaling pathway. RAW 264.7 cells were treated with various doses of L-quebrachitol and 2.5 µM BAY11-7082 in the presence of RANKL 20 ng/mL and M-CSF 20 ng/mL for 3 days. (**A**) Relative mRNA levels of NF-κB-P65, (**B**) NFATc1, and (**C**) c-Fos were evaluated using quantitative RT-PCR analysis. The data was obtained from 3 independent experiments on 4 replicates and presented as mean ± SEM. Letters on each column represent statistically significant differences at *p* < 0.05.

We investigated the role of the NF-κB signaling pathway induced by RANKL in osteoclast differentiation. The expression of NF-κB-P65, a component of NF-κB, was analyzed. The results showed that RANKL and M-CSF stimulation significantly increased NF-κB-P65 mRNA expression, which was effectively downregulated by L-quebrachitol treatment in a dose-dependent manner (Figure 5A). Additionally, the inhibitory effect of L-quebrachitol on osteoclast differentiation was confirmed by incubating the cells with an NF-κB inhibitor. Furthermore, we analyzed the expression of mRNA of NFATc1 and c-Fos, which are important regulators of osteoclastogenic genes. RANKL and M-CSF stimulation upregulated NFATc1 and c-Fos mRNA levels, while L-quebrachitol dose-dependently downregulated the expression of these genes. L-Quebrachitol at 60 µM showed the maximum inhibition of NFATc1 and c-Fos gene expression (Figure 5B,C), demonstrating the inhibitory effect of L-quebrachitol on NF-κB-P65, NFATc1, and c-Fos gene expression.

### 3.6. Effect of L-Quebrachitol on Osteoclastogenic Genes

**Figure 6:**
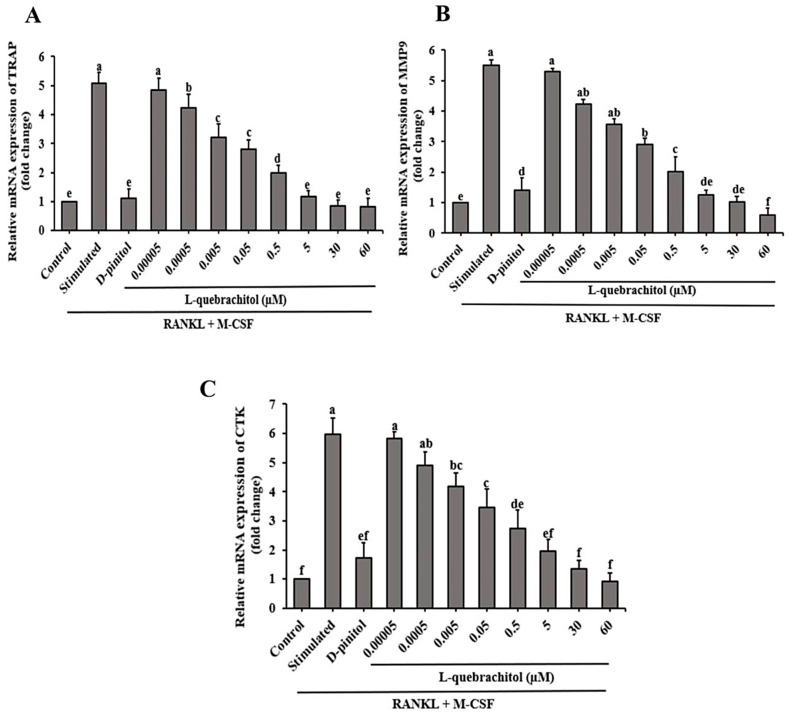
L-Quebrachitol downregulates the expression of osteoclastogenic genes. RAW 264.7 cells were treated with different doses of L-quebrachitol in the presence of RANKL (20 ng/mL) and M-CSF (20 ng/mL) for three days. (**A**) Relative mRNA levels of TRAP, (**B**) MMP-9, and (**C**) cathepsin K were evaluated using RT-PCR analysis. The data was obtained from 3 independent experiments on 4 replicates and presented as mean ± SEM. Letters on each column represent statistically significant differences at *p* < 0.05.

To additionally investigate the effect of L-quebrachitol on osteoclast generation, we determined the mRNA expression of digestive enzymes, such as TRAP, MMP-9, and cathepsin K. These enzymes are crucial for the collagenolytic ability of osteoclasts and their ability to resorb bone. qRT-PCR analysis showed that L-quebrachitol significantly and dose-dependently reduced the expression of TRAP, MMP-9 and cathepsin K, compared with the RANKL-stimulated group (Figure 6A–C).

### 3.7. Effect of L-Quebrachitol on Protein Expression Level of NF-κB-P65, NFACTc1, and C-Fos

**Figure 7:**
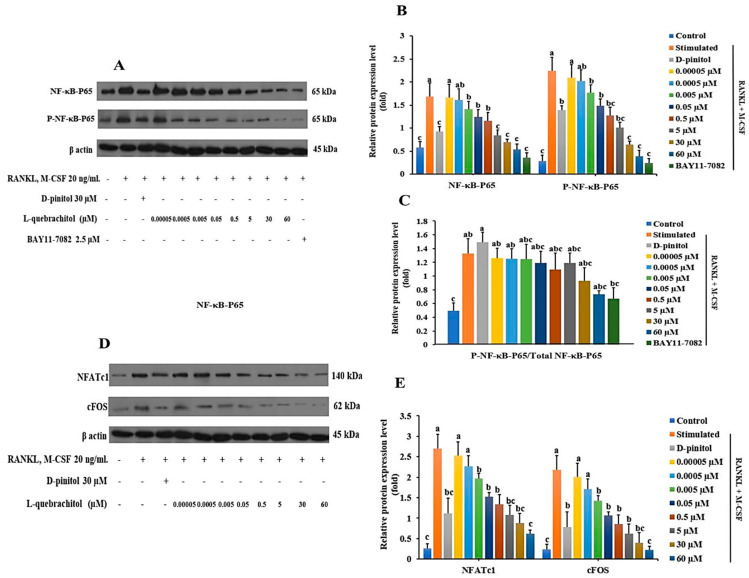
L-Quebrachitol downregulates the protein expression of master transcription factors in osteoclasts (RAW 264). Briefly, cells were treated with different doses of L-quebrachitol or BAY11-7082 in the presence of RANKL (20 ng/mL) and M-CSF (20 ng/mL) for three days. Protein expression levels of (**A**) NF-κB-P65, P-NF-κB-P65, and (**D**) NFATc1 and c-Fos were evaluated using Western blot analysis, and (**B**,**C**,**E**) the protein intensity was quantified using ImageJ. The data was obtained from 3 replicates and presented as mean ± SEM. Letters on each column represent statistically significant differences at *p* < 0.05.

The RANKL/RANK/NF-κB signaling pathway is a crucial mechanism in osteoclastogenesis. The results of the Western blot analysis indicated that L-quebrachitol dramatically reduced the phosphorylation of NF-κB-P65 induced by RANKL (Figure 7A–C). Similar inhibition of NF-κB-P65 phosphorylation was observed in the group treated with BAY11-7082 (NF-κB inhibitor), which further confirmed the suppressive ability of L-quebrachitol on NF-κB-P65 (Figure 7A,C). Furthermore, L-quebrachitol dose-dependently suppressed the protein levels of NFATc1 and c-Fos (Figure 7D,E). These findings demonstrate that L-quebrachitol attenuates the protein expression of NF-κB-P65, NFATc1, and c-Fos, the crucial key transcription factors in osteoclasts.

### 3.8. Effect of L-Quebrachitol on the Expression of RANK

**Figure 8:**
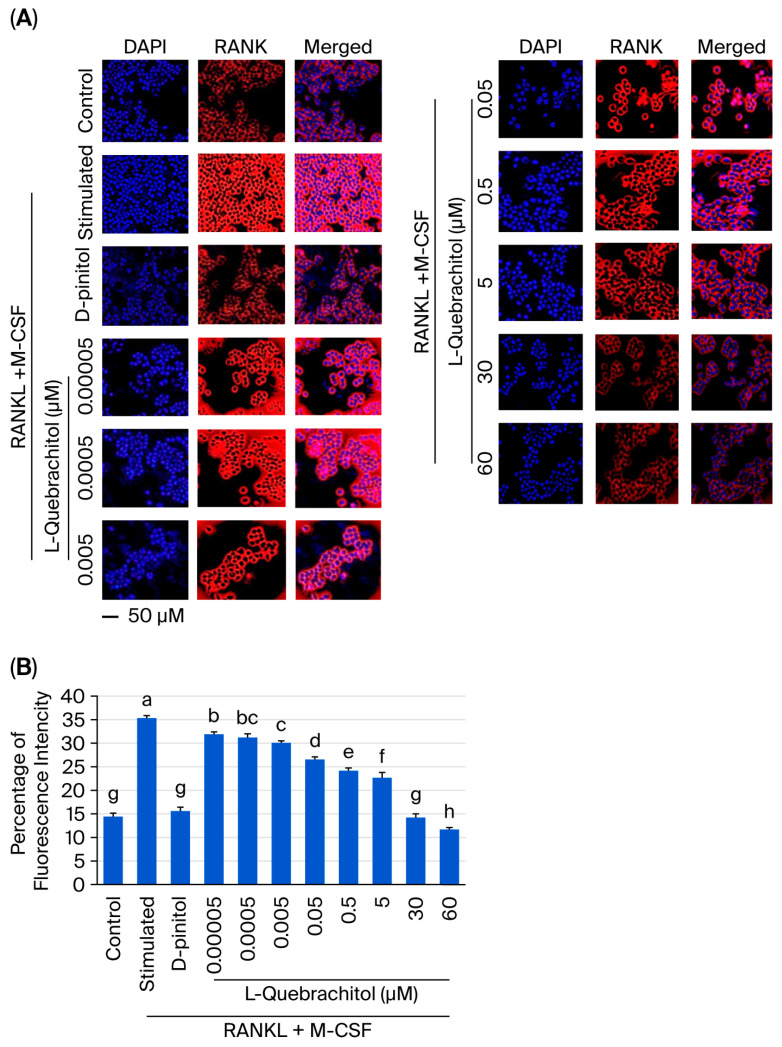
L-Quebrachitol remarkably downregulates the expression of RANK. RAW 264.7 cells were treated with different doses of L-quebrachitol in the presence of RANKL (20 ng/mL) and M-CSF (20 ng/mL) for 24 h. (**A**) Expression of RANK was measured via immunofluorescence staining; the fluorescence image was captured with a fluorescence microscope at a magnification of 20× and (**B**) analyzed using Image J software. The data was obtained from 3 independent experiments in 4 replicates and presented as mean ± SEM. Letters on each column represent statistically significant differences at *p* < 0.05.

RANK is a crucial receptor that binds with RANKL, and their interaction recruits the adaptor protein TRAF 6, leading to the activation of various signaling pathways essential for osteoclastogenesis, such as NF-κB and MAPK [31]. We performed immunofluorescence staining to determine the effect of L-quebrachitol on RANK expression. As displayed in Figure 8A,B, RANK expression was markedly induced by RANKL after 24 h of induction. We investigated whether L-quebrachitol could inhibit the RANKL-induced expression of RANK. Our results showed that L-quebrachitol suppressed RANK expression in a dose-dependent manner (Figure 8A,B). These findings denote that L-quebrachitol can attenuate the RANK expression, which is a crucial receptor for activating the signaling cascades of osteoclastogenesis.

### 3.9. Effect of L-Quebrachitol on Differentiated Protein Expression

**Figure 9:**
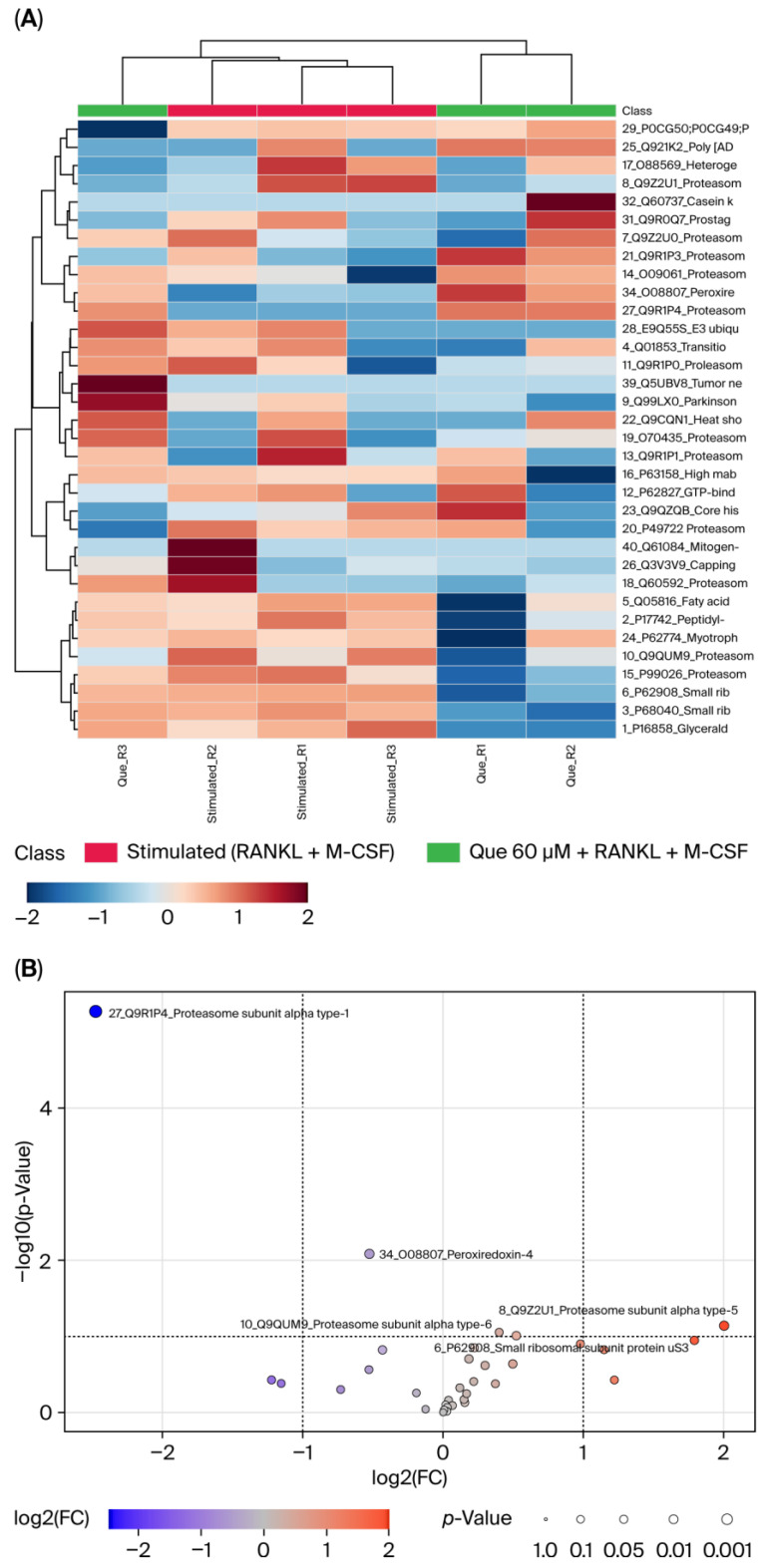
Effect of L-Quebrachitol on protein expression profiling. RAW 264.7 cells were treated with L-quebrachitol at 60 µM in the presence of RANKL (20 ng/mL) and M-CSF (20 ng/mL) for 72 h, and then LC-MS analysis was performed. (**A**) Heat map and (**B**) Volcano plot (stimulated/L-quebrachitol 60 µM) of L-quebrachitol-treated cells in 3 replicates.

A proteomic assay was performed to further investigate the effect of L-quebrachitol on differentiated protein expression. It was observed that many proteins were affected by compound such as peroxiredoxin-4 (PRDX4), small ribosomal subunit protein US3 (US3), proteasome subunit alpha type-1 (PSMA1), proteasome subunit alpha type-6 (PSMA6), and particularly proteasome subunit alpha type-5 (PSMA5), which was clearly downregulated by L-quebrachitol, associated with NF-κB activation (Figure 9A,B).

### 3.10. Effect of L-Quebrachitol on Ovariectomized Rats

**Figure 10:**
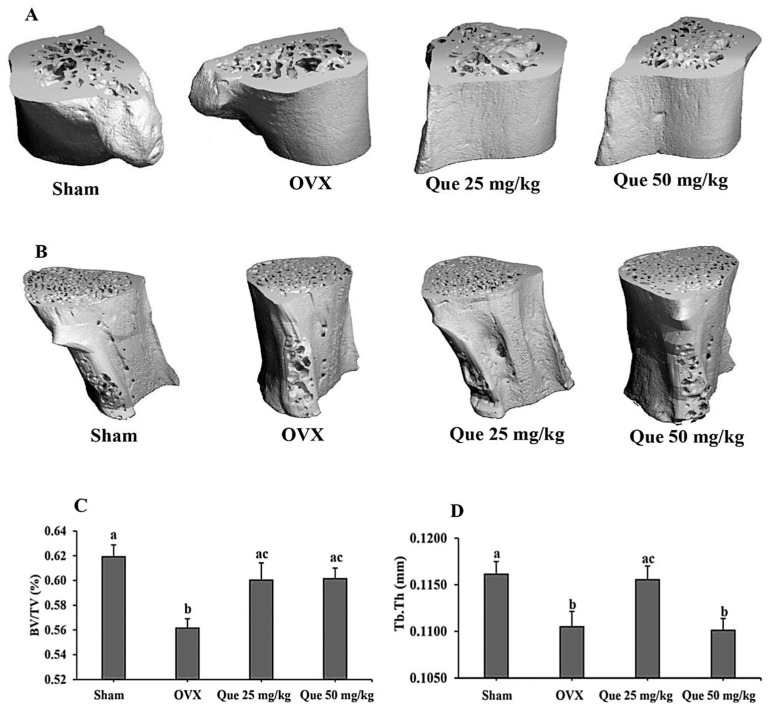
Effect of L-quebrachitol on ovariectomized model. After the rats were given L-quebrachitol for twelve weeks, the femur and lumbar bone were collected for Micro-CT analysis at 55 kVp, 145 A, and 221 ms on a Scanco CT80 scanner. Micro-CT images of (**A**) femur (left) and (**B**) lumbar (L5 vertebrae). (**C**) BV/TV of femur bone and (**D**) Tb.Th of lumbar bone. Group: Sham, N = 5; OVX, N = 5; Que 25 mg/kg, N = 5; Que 50 mg/kg, N = 5. The data are presented as mean ± SEM. Letters on each column represent statistically significant differences at *p* < 0.05.

The ovariectomized model was used as a model of postmenopausal status, a risk factor of osteoporosis [4]. Serum estradiol concentrations were evaluated and showed a marked decrease in the OVX group. Both the ovariectomized control and the treatment groups exhibited serum estradiol levels below 5 pg/mL, whereas the sham group demonstrated significantly higher levels at 28.94 ± 4.68 pg/mL. For detection of L-quebrachitol on bone parameters, the femur and lumbar bone were gathered for Micro-CT testing to detect the BV/TV and Tb.Th value. The results clearly showed that the compound could significantly reduce bone loss and enhance both parameters compared with the OVX model, supporting the potential of L-quebrachitol in preventing bone loss (Figure 10A–D).

## 4. Discussion

Inositol, a sugar alcohol, plays a crucial role in the physiological system. Two major stereoisomers of inositol, Myo-inositol and D-chiro inositol, are distributed in the human body. Myo-inositol plays a vital function in cell membranes, where it is found in the form of phosphatidyl-myo-inositol. Additionally, it serves as a secondary messenger that regulates hormonal activities, including insulin, follicle-stimulating hormone, and thyroid-stimulating hormone [32,33,34]. Normally, inositol does not act directly but interacts with several biomolecules, such as sphingolipids, inositol pyrophosphates, and inositol methyl ethers [16,35,36]. In particular, methyl-inositol has been shown to exhibit anti-inflammatory, anticancer, and antiosteoporosis activities [19,20]. According to a prior study, D-pinitol, a methyl derivative of D-chiro-inositol, attenuates osteoclast differentiation by interrupting the NF-ƘB and MAPK signaling pathways [22]. Apparently, most isomers exhibit significant differences in effects. Consequently, one isomer may provide the desired therapeutic efficacy, while the other may be inactive [28]. Accordingly, our previous research demonstrated that L-quebrachitol promotes osteoblast proliferation, differentiation, and mineralization [29], whereas D-pinitol shows no modulatory effect on osteoblasts. However, the effect of L-quebrachitol on osteoclast formation remains unclear. Hence, this research is the first to report that L-quebrachitol can potentially suppress osteoclast differentiation and function. Quantitative purity assessment using an external calibration curve demonstrated that purified L-quebrachitol exceeded 98% purity. This level of purity is comparable to that reported by Li et al. (2023), who achieved approximately 99% purity following the isolation and purification of L-quebrachitol from natural rubber industry wastewater [30]. In addition, cell viability assays showed that at concentrations lower than 500 µM, no cytotoxic effects were observed on the cells at any time.

Several collagenolytic enzymes, such as MMP-9, TRAP, and cathepsin K, have been used to evaluate the maturation of osteoclasts. Among these, TRAP is widely used as a crucial histochemical marker of osteoclast maturity [37,38]. TRAP staining results showed that L-quebrachitol significantly suppressed multinucleated cells, compared with that in the RANKL-stimulated group, consistent with the positive control group treated with D-pinitol, which has also been shown to inhibit the number of TRAP-positive cells. This result confirms that L-quebrachitol can attenuate the fusion of mature osteoclasts, implying that the bone resorption function of osteoclasts is likely to be reduced.

During the resorption stage, osteoclasts adhere to the surface of bone and initiate the resorption process by creating an acidic environment and secreting digestive enzymes. Thereafter, they leave behind resorption lacunae or pit formation areas [37,39,40]. Hence, the resorption level in these areas can represent the bone digestion ability of mature osteoclasts. The pit formation assay revealed that L-quebrachitol significantly and dose-dependently reduced the pit formation areas compared with those in the stimulation group. Furthermore, the results were similar to those in the D-pinitol-treated group, which demonstrated significantly suppressed pit formation. This result confirms that L-quebrachitol not only suppresses osteoclast formation but also potentially attenuates osteoclast resorption ability. The decline in osteoclast activity may be attributed to the decreased levels of proteolytic enzymes, including TRAP, MMP-9, and cathepsin K, which play a significant role in the digestion of the bone matrix by osteoclasts.

In this study, expression of NF-κB-P65 was evaluated at both the protein and mRNA levels. The results showed that NF-κB-P65 expression was dose-dependently decreased, consistent with a recent study that showed that a rare sugar, D-allose, could strongly inhibit TRAP-positive cells through the NF-κB pathway [41]. The proteomic assay found strongly suppressed expression of PSMA5, a component of the 20S proteasome consisting of seven different α (PSMA1-7) and β (PSMB1-7) subunits, which play a crucial role in intracellular protein degradation and indirectly impact NF-κB activation through the degradation of NF-κB inhibitor IκBα [7,42,43]. Furthermore, after treatment with another NF-κB inhibitor, BAY11-7082, the expression level distinctly decreased, confirming the suppressive effect of L-quebrachitol on the NF-κB canonical cascade.

NFATc1 and c-Fos are well-established as key transcription modulators involved in osteoclast differentiation and are regulated by NF-κB. The crucial function of NFATc1 in osteoclastogenesis has been proven by various studies conducted on genetically modified mutant mice [44]. Osteoclast precursor cells inserted with NFATc1 could induce osteoclast formation in these cells, in spite of the lack of RANKL [45]. Furthermore, NFATc1-knockout mice reportedly exhibit osteoclast differentiation failure and develop an osteoporotic bone phenotype [44,46]. The beginning of the osteoclast lineage from monocyte/macrophage precursors is enhanced mainly by RANK trimerization, and NFATc1 stimulation, as well as transcription modulators such as NF-κB and c-FOS, induces the gene expression of factors necessary for osteoclast activation, including TRAP, cathepsin K, and MMP-9 [45,47,48]. During maturation, osteoclasts go through morphological change forming a multinucleated cell, and NFATc1 and c-Fos govern the expression of fusion-related molecules such as dendritic cell-specific transmembrane protein (DC-STAMP) and osteoclast stimulatory transmembrane protein (OC-STAMP) [49,50]. Hence, we investigated the effect of L-quebrachitol on NFATc1 and c-Fos expression levels. Our results demonstrated that L-quebrachitol significantly reduced the expression of both NFATc1 and c-Fos at both the transcriptional and protein levels. Additionally, the results also revealed a reduction in osteoclast marker genes TRAP, cathepsin K, and MMP-9, leading to the inhibition of osteoclast formation and bone resorption activity. This suggests that L-quebrachitol can exert a negative effect on osteoclast differentiation by attenuating master modulators such as NFATc1 and c-Fos.

Consistent with previous findings, D-chiro-inositol strongly suppressed multinucleated osteoclast formation and bone resorption activity through the attenuation of the NF-κB/NFATc pathway and proteolytic enzymes such as TRAP and cathepsin K [51]. Furthermore, an in vivo study demonstrated that D-pinitol significantly decreased bone loss in ovariectomized mice by suppressing NF-κB-P65 activation [22].

A previous study demonstrated the suppressive effect of inositol hexakisphosphate (IP6) on osteoclast activity by reducing the expression of digestive enzymes [52]. Consistent with this, L-quebrachitol significantly downregulated the expression of proteolytic enzymes TRAP, MMP-9, and cathepsin K, which are crucial for bone resorption by osteoclasts. This was also consistent with the observed reduction in pit formation areas.

The RANK/RANKL/OPG system is a critical mechanism that regulates bone remodeling and osteoclastogenesis. The initial binding of RANK to its receptor triggers many downstream signaling pathways essential for osteoclast differentiation. However, OPG, a decoy receptor, can also bind with RANKL and inhibit osteoclastogenesis; this mechanism is naturally occurring in the regulation of osteoclastogenesis [53,54]. Therefore, RANK could be the primary target of L-quebrachitol to suppress osteoclast differentiation. In this regard, the RANK expression level was subsequently detected by immunofluorescence staining. As expected, the expression of RANK was significantly induced by RANKL after induction for 24 h. However, L-quebrachitol treatment led to a concentration-dependent inhibition of its expression level, demonstrating that L-quebrachitol could significantly reduce RANK expression. This reduction might result in a decrease in the interaction between RANK and RANKL, leading to the inhibition of many osteoclastogenic modulators, including NF-κB-P65, NFATc1, and c-Fos, as shown in the Western blot analysis. This finding correlates with previous results on other candidate therapeutic agents such as Necrostatin-7 and Decitabine, which have demonstrated suppressive activities on osteoclastogenesis through the RANK/NF-κB/NFATc1 pathway and prevented bone loss in ovariectomized mice [55,56].

The OVX model of osteoporosis imitates estrogen deficiency-induced bone deprivation and shows clinical manifestations of postmenopausal osteoporosis [57]. Female sex hormones are thought to be key regulators of bone metabolism and are considered primary risk factors in the development of bone-related diseases. Osteoblasts, osteoclasts, and osteocytes possess functional estrogen receptors (ERs), through which estrogen exerts significant influence on bone formation, resorption, and overall skeletal homeostasis [58]. Previous studies have reported that a decrease in estrogen as a result of menopause or ovariectomy is related to enhanced secretion of IL-6, IL-1, TNFa, and RANKL from osteoblasts or peripheral blood monocytes, which reduces expression of TGFβ in the bone and as a result promotes osteoclastogenesis [59]. Furthermore, estrogen has been found to induce apoptosis of murine osteoclast-like cells through TGFβ [60]. The investigation evaluated the long-term effects of estrogen deficiency resulting from ovariectomy by quantifying serum estradiol concentrations. The OVX rats demonstrated a marked reduction in serum estrogen levels relative to the sham group. This outcome demonstrated the effect of estrogen level modulations in OVX rats and confirmed the validity of our results and interpretations. Our results show that L-quebrachitol at a low dose (25 mg/kg) significantly increased the BV/TV and Tb.Th values in ovariectomized rats, whereas a recent study demonstrated that L-quebrachitol at a dose of 125 mg/kg⋅bw could significantly increase mineral density and calcium content in the bones of ovariectomized rats [30]. A previous study also reported that a decrease in estrogen induced osteoblasts to produce RANKL, which is the regulator of osteoclasts [61]. Meanwhile, our prior study reported that L-quebrachitol could reduce the synthesis of RANKL by osteoblasts [29], which was related to the reduction in mature osteoclasts in TRAP staining. Moreover, proteolytic enzymes involved in bone matrix degradation, including TRAP, CTK, and MMP-9, were all inhibited leading to reduced osteoclast bone resorption activity, as shown in the pit formation assay. Taken together, the results of both the RAW 264.7 cell and animal models supported the anti-bone loss property of L-quebrachitol. Therefore, this investigation provided strong evidence on the effect of L-quebrachitol on bone metabolic homeostasis. These results suggest the potential of L-quebrachitol as a therapeutic approach for osteoporosis treatment.

## 5. Conclusions

In conclusion, this investigation is the first to report the ability of L-quebrachitol to inhibit osteoclast formation and decrease osteoclast resorption, possibly through the inhibition of the RANKL-induced NF-κB signaling pathway, by inhibiting NF-κB-P65, NFATc1, and c-Fos expression, along with inhibiting bone digestive enzymes such as TRAP, cathepsin K, and MMP-9. L-quebrachitol also reduces the expression of RANK as well as PSMA5. Additionally, L-quebrachitol could increase the BV/TV and Tb.Th values of ovariectomized rats. Thus, these findings not only contribute to a better understanding of the underlying molecular mechanism of L-quebrachitol in osteoclastogenesis but also suggest that L-quebrachitol has the potential to inhibit bone resorption while promoting bone formation; this would create value for *Hevea brasiliensis* latex serum, an abundant source of L-quebrachitol that is largely discarded by the rubber industry.

## Figures and Tables

**Table 1:** Primer sequences of qRT-PCR.

Gene	Sequence	GenBank Accession No.
NF-κB-P65	F: TCACCGGCCTCATCCACAT	XM_006531694.4
	R: TGGCTAATGGCTTGCTCCAG	
NFATc1	F: CACACACCCCGCATGTCA	NM_001164110.1
	R: CGGGCCGCAAAGTTTCTC	
c-Fos	F: AGCTCCCACCAGTGTCTACC	NM_010234.3
	R: TCACCGTGGGGATAAAGTTGG	
TRAP	F: TGGATTCATGGGTGGTGCTG	XM_006509946.3
	R: CGTCCTCAAAGGTCTCCTGG	
MMP-9	F: CTCTGCTGCCCCTTACCAG	NM_013599.5
	R: CACAGCGTGGTGTTCGAATG	
Cathepsin K	F: AGTAGCCACGCTTCCTATCC	NM_007802.4
	R: GAGAGGCCTCCAGGTTATGG	
GAPDH	F: AGGTCGGTGTGAACGGATTTG	XM_036165840.1
	R:TGTAGACCATGTAGTTGAGGTCA	

## Data Availability

The original contributions presented in this study are included in the article/Supplementary Material. Further inquiries can be directed to the corresponding author.

## Supplementary Materials

The following supporting information can be downloaded at: https://www.mdpi.com/article/10.3390/biom16010168/s1, Figure S1: Effect of L-quebrachitol on the expression of NFκB-P65, P-NFκB-P65 and β-actin represents western blot analysis shown in Figure 6A; Figure S2: Effect of L-quebrachitol on the expression of NFATc1 and cFOs; represents western blot analysis shown in Figure 6C.
