# Supplementary material for: L-Quebrachitol Attenuates RANKL-Induced Osteoclastogenesis and Bone Resorption in Ovariectomized Rat Model
Source: Biomolecules. 2026 Jan 20;16(1):168. doi: 10.3390/biom16010168 (PMC12838629; doi:10.3390/biom16010168)
Supplement: Supplementary file 1 [file biomolecules-16-00168-s001.zip › biomolecules-4044139-supplementary.pdf]

Original western blot analysis of L-quebrachitol for three repeats

The effects of L-quebrachitol on the expression of osteoclastogenesis-related protein including NFATc1, cFOS, NFƘB-P6, P-NFƘB-P65 and β-actin were detected with western blot.

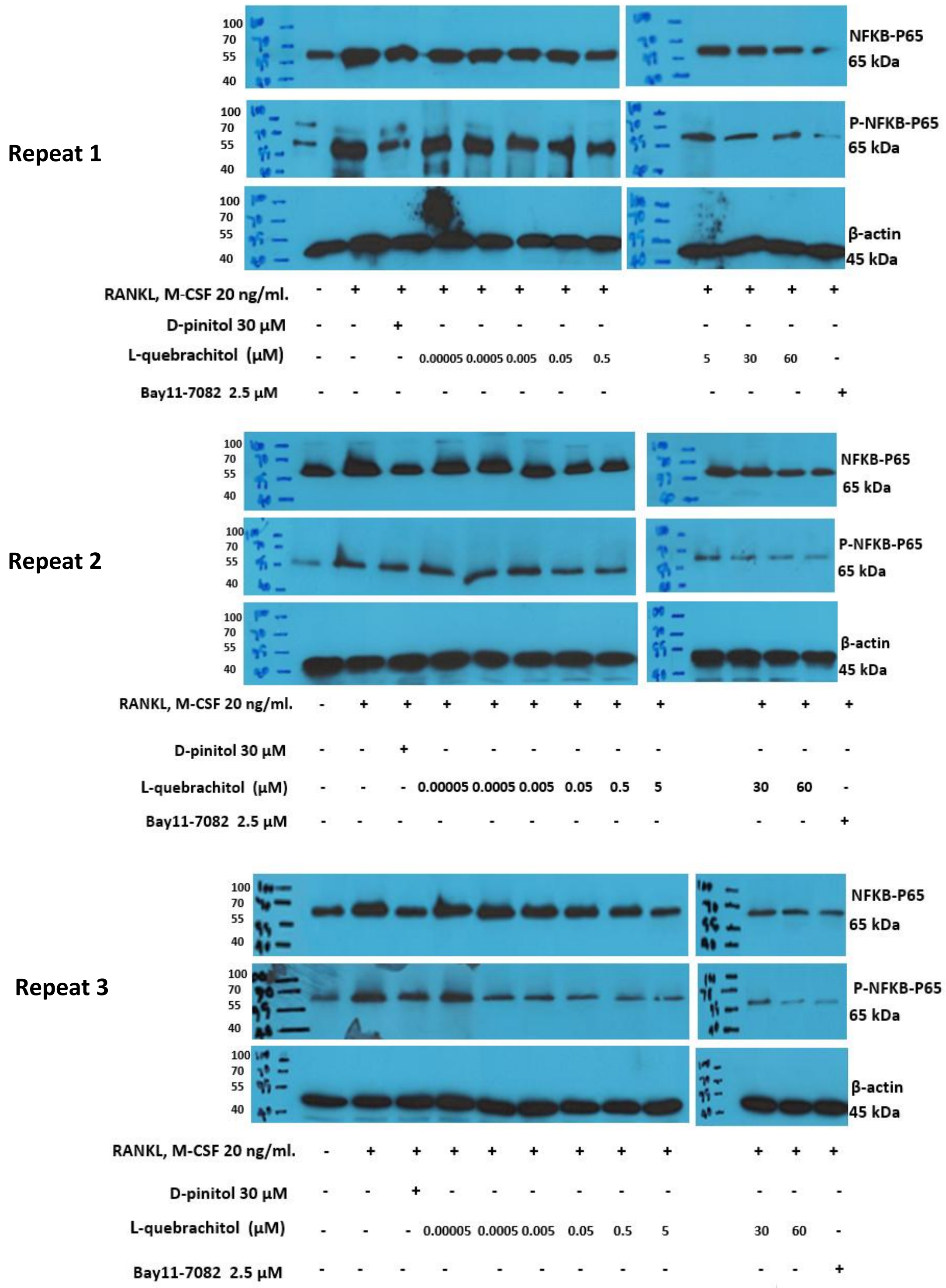

Figure S1. Effect of L-quebrachitol on the expression of NFƘB-P65, P-NFƘB-P65 and β-actin; represents western blot analysis shown in **Fig 6A**

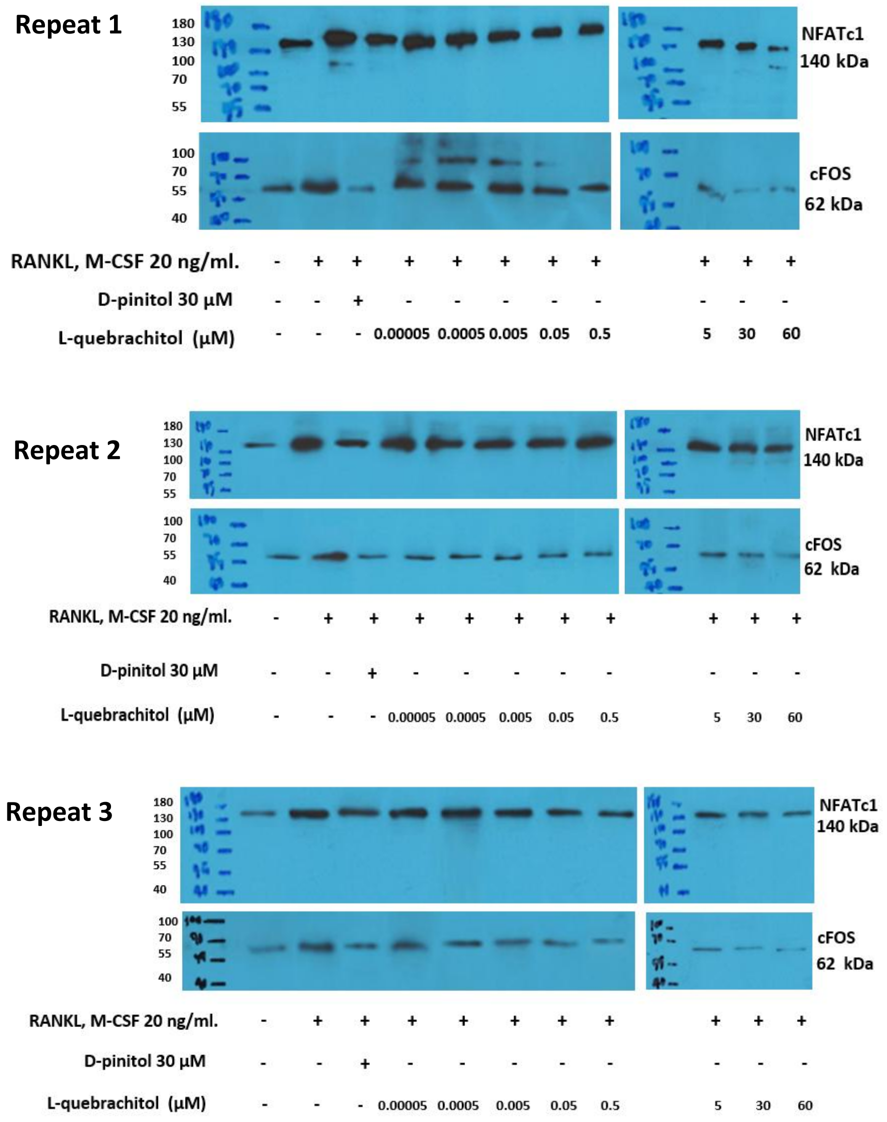

Figure S2. Effect of L-quebrachitol on the expression of NFATc1 and cFOs; represents western blot analysis shown in **Fig 6C**
